# Influence of Coexisting Copper and Zinc on the Adsorption and Migration of Sulfadiazine in Soda Saline–Alkali Wetland Soils: A Simulation Approach

**DOI:** 10.3390/molecules31010189

**Published:** 2026-01-05

**Authors:** Wencong Yang, Xia Wu, Wenyue Shao, Nana Luo, Jia Zhou

**Affiliations:** 1 School of Geographical Sciences, Harbin Normal University, Harbin 150025, China; ywc@stu.hrbnu.edu.cn (W.Y.); wx98926417@163.com (X.W.); sh1953594466@163.com (W.S.); 2 School of Geomatics and Prospecting Engineering, Jilin Jianzhu University, Changchun 130118, China

**Keywords:** sulfadiazine, heavy metals, soils of soda saline–alkali wetlands, adsorption, migration

## Abstract

This study investigates the adsorption and migration of sulfadiazine (SDZ) in soda saline–alkali soils under Cu/Zn co-pollution using equilibrium adsorption and soil column experiments. Freundlich and Langmuir isothermal models, combined with Hydrus-1D two-site modeling, revealed concentration-dependent interactions. Low Cu (10–100 mg kg^−1^) and Zn (10–100 mg kg^−1^) enhanced SDZ adsorption via charge regulation and complexation, while high concentrations (300 mg kg^−1^) suppressed adsorption through competitive adsorption and hydroxide precipitation. Synergistic Cu-Zn coexistence further reduced adsorption to 3.035 mg kg^−1^. Freundlich modeling (R^2^ = 0.922–0.995) outperformed Langmuir, confirming adsorption site heterogeneity. Column experiments showed Cu (300 mg kg^−1^) and Zn (300 mg kg^−1^) accelerated SDZ migration (peaks 0.93–0.94), delaying breakthrough versus Br^−^. Hydrus-1D simulations (R^2^ ≥ 0.915, RMSE < 0.1) effectively quantified nonlinear dynamics between instantaneous adsorption sites (f = 0.101–0.554) and metal concentrations. Results demonstrate heavy metals critically regulate antibiotic fate via concentration-dependent mechanisms in saline–alkali ecosystems.

## 1. Introduction

In recent years, antibiotics, as an emerging type of pollutant, have been extensively applied in different fields such as human medical treatment, aquaculture, animal husbandry, and agriculture. Owing to their low bioavailability, antibiotics cannot be fully metabolized by the human body or animals, leading to their eventual discharge into water and soil environments [1,2,3,4]. At present, the co-occurrence of heavy metal and antibiotic pollution has become a research hotspot. Andreu found a significant correlation between antibiotics and heavy metals in the wetland waters along the Mediterranean coast [5]. This discovery is of great significance for understanding the pollution status of wetland ecosystems. It implies that in the wetland environment, antibiotics and heavy metals do not exist independently but are interconnected and interact with each other. Therefore, as a gathering place for environmental pollutants, wetland soil is also one of the main destinations for heavy metals.

Sulfonamides, characterized by their low cost, remarkable curative effect, low toxicity, and wide applicability, are frequently used in the livestock and poultry breeding industry [6,7]. As a typical sulfonamide, sulfadiazine (SDZ) has a pKa ≈ 6.5. It is easily dissociated into anions under alkaline conditions, while Cu^2+^ or Zn^2+^ is prone to form hydroxyl complexes (e.g., Cu(OH)_2_) at a high pH. These interactions may influence the adsorption-migration behavior of SDZ via electrostatic forces or coordination bonds [8,9]. The coexistence of Copper (Cu) and Zinc (Zn) may either enhance or inhibit the processes of adsorption, desorption, and migration of SDZ within the soil matrix [10,11]. Yang, L.-L demonstrated that the combined pollution of Cu and sulfamethoxazole could pose toxicity to wetland plants, leading to changes in the structure of the microbial community [12]. However, most existing studies focus on the environmental behavior of single pollutants, while the mechanisms underlying the combined pollution of SDZ, Cu, and Zn in wetland soils warrant further investigation. Sulfonamide antibiotics, including sulfadiazine (SDZ), have been widely detected in surface waters, soils, sediments, and groundwater, with reported concentrations ranging from ng L^−1^ to μg L^−1^ in aquatic environments and μg kg^−1^ levels in soils, especially in areas influenced by intensive livestock farming. These compounds mainly enter the environment through livestock manure application, wastewater treatment plant effluents, aquaculture activities, and irrigation with contaminated water [13,14,15,16,17].

The fate of SDZ and heavy metals in the soil depends on the movement of water flow and solutes. In turn, the movement of water flow and solutes is governed by the hydraulic properties of the soil, as well as the physicochemical processes occurring in the soil profile. Currently, the Hydrus-1D software is used to study the migration of pollutants. The two-site model in the Hydrus-1D software considers the mobile and immobile water regions and can be used to explain the migration of pollutants. This model incorporates the adsorption of pollutants at different adsorption sites. For example, the adsorption at type I adsorption sites is instantaneous, while the adsorption at type II adsorption sites changes over time. This model has been widely applied across various disciplines, including soil science, environmental science, and hydrogeology [18,19,20]. Wu Y. coupled the dynamic Level IV fugacity model with the Hydrus-1D model to estimate the fate and transport of sulfamethoxazole in water and soil in Tianjin City, a water-scarce city, China [21]. Li studied the migration law of florfenicol in purple soil based on Hydrus-1D [22].

Soda saline–alkali wetlands are typically characterized by high ecological vulnerability and sensitivity, serving as critical ecological barriers [23]. When improvement measures such as “growing rice to decrease soil alkalinity” and applying organic fertilizers are implemented in the surrounding saline–alkali dry farmlands, agricultural activities like water diversion for irrigation, fertilization, and the use of fungicides impact the ecological environment. The drainage water from farmlands is an important recharge source for wetlands, but it contains many residual antibiotics, Cu and Zn [24,25]. Due to moderate salinization and alkalization of soils in soda saline–alkali wetlands, these soils exhibit low fertility, insufficient organic matter content, and weak pollutant adsorption capacity. Moreover, the self-purification capacity of wetland water bodies is limited. As a result, the antibiotics and heavy metals carried by farmland drainage water are challenging to degrade or dilute effectively, facilitating their accumulation and migration within the wetland system. This process exacerbates the degradation of the living environment for wetland organisms and water pollution. Recent studies have reported the co-occurrence of antibiotics and heavy metals in soils and wetland environments and demonstrated that metals such as Cu, Zn, Cd, and Pb can regulate the adsorption and mobility of sulfonamide antibiotics through electrostatic interactions, surface complexation, and competitive adsorption, with strong dependence on pH, soil properties, and metal loading [26,27]. However, most existing studies focus on agricultural soils or aquatic and constructed wetland systems, while systematic investigations targeting soda saline–alkali wetland soils under alkaline–saline conditions remain limited. Therefore, this study integrates batch adsorption experiments, soil column migration experiments, and Hydrus-1D two-site modeling to investigate the coupled adsorption–migration behavior of sulfadiazine under Cu and Zn co-contamination in a typical soda saline–alkali wetland soil. The results quantitatively reveal the concentration-dependent and synergistic effects of Cu and Zn on sulfadiazine retention and transport, providing new insights into antibiotic fate under complex geochemical conditions and supporting the prediction and management of antibiotic migration risks in saline–alkali wetland ecosystems. Unlike previous Cu-Zn-antibiotic studies focused mainly on neutral agricultural soils or aquatic systems, this work reveals concentration-dependent and synergistic metal effects on antibiotic behavior under alkaline–saline wetland conditions.

Based on these considerations, we hypothesize that (1) the adsorption and migration of sulfadiazine in soda saline–alkali wetland soils are strongly controlled by soil alkalinity and salinity, and (2) coexisting Cu and Zn exert concentration-dependent and synergistic effects on sulfadiazine retention and transport by altering adsorption site distribution and metal–antibiotic interactions.

The research objectives of this paper are as follows: (1) to explore the effects of different concentrations of Cu and Zn on the isothermal adsorption of SDZ, (2) to clarify the migration of the tracer Br^−^ in the soil column, (3) to analyze the migration characteristics of SDZ under different concentrations of Cu and Zn, (4) and to clarify the changes in the parameters of the breakthrough curve of SDZ in the presence of different concentrations of Cu and Zn. Through adsorption experiments, migration studies, and model simulations, hydrodynamic parameters, solute transport parameters, and inversion parameters can be obtained. These parameters not only quantitatively characterize the interaction between SDZ and soil but also provide insights into the retention or leaching behavior of SDZ in soil, thereby facilitating a deeper and more comprehensive understanding of its environmental fate.

## 2. Results and Discussion

### 2.1. Effects of Different Cu and Zn Concentrations on the Isothermal Adsorption of SDZ

**Figure 1:**
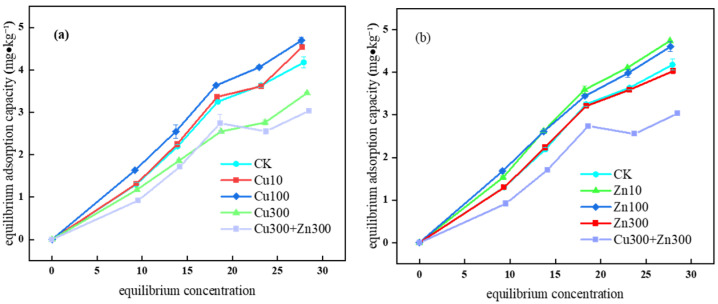
Adsorption isotherms of sulfadiazine in soda saline–alkali wetland soil under different Cu and Zn concentrations: (**a**) Adsorption isotherms under different Cu concentrations; (**b**) Adsorption isotherms under different Zn concentrations.

As the equilibrium concentration of SDZ increased, the adsorption capacity of the soda saline–alkali wetland soil increased in all treatments (Figure 1). At a low SDZ concentration, the adsorption sites on the soil surface were sufficient, yielding remarkable adsorption effects. As the SDZ concentration increased, the adsorption capacity gradually leveled off. Meanwhile, different concentrations of Cu and Zn significantly affected SDZ adsorption capacity. As shown in Figure 1a, the SDZ adsorption capacities of Cu treatments and CK rank as Cu100 (4.69 mg kg^−1^) > Cu10 (4.54 mg kg^−1^) > CK (4.18 mg kg^−1^) > Cu300 (3.45 mg kg^−1^). Thus, with the increase in Cu concentration, the post-equilibrium SDZ adsorption capacity increased first and then decreased. The soil treated with Cu100 showed the highest SDZ adsorption capacity, while the soil treated with Cu300 showed the lowest. The results suggest that Cu (10–100 mg kg^−1^) rendered the adsorption sites more effective by altering the soil surface charge and forming Cu-OH complexes, thus promoting SDZ adsorption. However, the increased Cu concentration of 300 mg kg^−1^ produced excessive Cu^2+^ that formed Cu(OH)_2_ precipitation and occupied the adsorption sites, inhibiting SDZ adsorption [28]. These results are consistent with the conclusions of Chen regarding the complex system of copper ions (II) and cefazolin [29]. Their study pointed out that the bridging effect of copper ions can significantly enhance cefazolin adsorption capacity. However, excessively high antibiotic concentrations led to decreased adsorption efficiency of copper ions.

As shown in Figure 1b, the SDZ adsorption capacities of Zn treatments and CK rank as Zn10 (4.74 mg kg^−1^) > Zn100 (4.60 mg kg^−1^) > CK (4.18 mg kg^−1^) > Zn300 (4.02 mg kg^−1^). The adsorption capacity first increased (Zn10–Zn100) and then decreased at Zn300. The SDZ adsorption capacity increased slightly in the Zn(10–100) treatment. However, as the Zn concentration increased further (300 mg kg^−1^), Zn^2+^ formed Zn(OH)_2_ precipitation under alkaline conditions [30], which competed with SDZ for adsorption sites, resulting in a continuous decrease in SDZ adsorption capacity.

With coexisting Cu and Zn, the SDZ adsorption capacity decreased to 3.04 mg kg^−1^, which was 13.49% lower than that under the 300 mg kg^−1^ Cu treatment (3.45 mg kg^−1^) and 14.18% lower than that under the 300 mg kg^−1^ Zn treatment (4.02 mg kg^−1^), respectively. Thus, the synergistic inhibitory effect of Cu and Zn on SDZ adsorption is significant. In the meantime, the synergistic competitive effect of Cu and Zn significantly intensified the competition for adsorption sites. This may be because the coexisting two-site model ions may form a ternary complex with SDZ through competitive coordination, weakening the electrostatic or coordination binding ability between SDZ and the soil [31,32]. The fitting result with the Freundlich model, K_f_, also confirmed the decreased adsorption capacity. Among the two-site model parameters, the proportion of instantaneous adsorption sites (f = 0.442) and the adsorption coefficient (K_d_ = 0.390) decreased. Hence, coexisting heavy metals reduce the instantaneous SDZ adsorption capacity and accelerate SDZ migration by inhibiting its kinetic adsorption. The synergistic inhibitory effect of Cu and Zn on the SDZ adsorption behavior demonstrates the nonlinear superposition effect of multiple metal ions on composite pollutants.

The pH level significantly affects the SDZ adsorption by sandy soil [33]. The pH of soda saline–alkali wetland soils is relatively high (7.85), which facilitates Cu^2+^ and Zn^2+^ to form hydroxide precipitates. Meanwhile, some ions form hydroxyl complexes through hydrolysis [34]. Through mono-dentate or bi-dentate coordination, these hydroxyl metal ions generate surface complexes that are difficult to desorb, thus reducing the effective adsorption sites for SDZ. In addition, soil organic matter and clay particles may also form stable complexes with Cu^2+^ and Zn^2+^, further affecting the SDZ adsorption behavior. Therefore, the Cu and Zn concentrations and their interactions significantly alter the SDZ adsorption capacity of the soda saline–alkali wetland soils. In recent years, the co-occurrence of antibiotics and metals in soils has been increasingly reported, and their interactions are now recognized as an important factor controlling pollutant fate rather than a simple additive effect. Recent reviews and experimental studies emphasize that antibiotics–metal co-contamination can alter adsorption competition, complexation behavior, and the distribution of reactive sites, thereby regulating mobility and environmental risks under different geochemical conditions. This perspective supports our observation that the Cu/Zn effects are concentration-dependent and can shift from promotion to inhibition when precipitation and site blocking become dominant under alkaline conditions [26].

Table 1 shows a higher fitting correlation coefficient of the Freundlich model (0.922–0.995) than the Langmuir model (NA-0.990), identifying the Freundlich model as more suitable for describing the SDZ adsorption behavior of the soda saline–alkali wetland soils. Moreover, the soil surface is heterogeneous, with a different distribution of adsorption sites and energy [35]. Zn100 showed the highest Freundlich constant (K_f_ = 0.276), representing the adsorption capacity, which suggested that the soda saline–alkali wetland soil had a relatively strong SDZ adsorption capacity for additional heavy metals. The adsorption intensity parameter 1/n in the Cu300 + Zn300 treatment (0.863) is higher than the Zn100 treatment (0.851), implying different adsorption mechanisms. The 1/n of all treatments is below 1, presenting an “L” type isotherm. Thus, SDZ initially exhibits a strong affinity with the soil surface, and the adsorption gradually approaches saturation with increased SDZ concentration. These results indicate that soil heterogeneity and competitive adsorption significantly affect the SDZ adsorption behavior of the soda saline–alkali wetland soils. In this study, the SDZ adsorption behavior is significantly affected by soil heterogeneity and competitive adsorption, similar to the findings of Conde-Cid, who identified a synergistic effect on TC and SDZ adsorption in crop soil and the effect of pH on SDZ adsorption [36]. Raquel identified the effects of agricultural soil texture and organic carbon content on SDZ mobility [37].

### 2.2. Tracer Br^−^ Migration in the Soil Column

**Figure 2:**
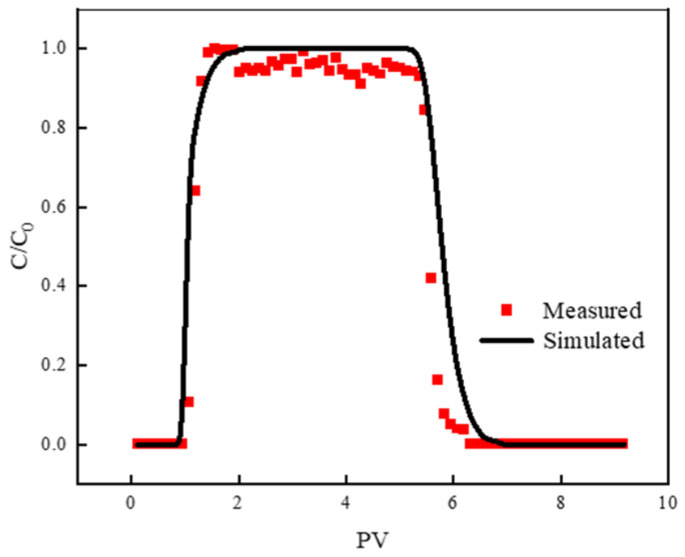
Measured and simulated breakthrough curves of Br^−^ in the soil column.

Br^−^, serving as an inert tracer, exhibits an adsorption coefficient of K_d_ = 0, indicating negligible Br^−^ adsorption in the soil. Therefore, the Br^−^ migration is dominantly through the convection-dispersion mechanism. To pinpoint the Br^−^ migration process in the soil and obtain the hydrodynamic parameters of the soil column, this study employed the classical convection-dispersion equation in Hydrus-1D to inverse model the breakthrough curve of Br^−^. The model assumes that the hydrodynamic dispersion coefficient (D) is mainly controlled by mechanical dispersion, with molecular diffusion considered negligible under the experimental flow conditions, and the dispersivity (λ) and the D value were calculated by fitting with experimental data (Table 2).model simulation results showed a relatively high fitting degree of the Br^−^ breakthrough curve (R^2^ > 0.900, RMSE < 0.1). Thus, the CDE model can accurately characterize the Br^−^ migration behavior in the soda saline–alkali wetland soil (Figure 2). The dispersion was relatively weak but non-negligible, consistent with the sandy texture of the soil. In addition, no low-concentration tailing phenomenon was observed in the breakthrough curve of Br^−^. Hence, the intraparticle diffusion within the soil profile can be neglected, further verifying the assumption of solute transport in a homogeneous porous medium. In this study, the breakthrough of Br^−^ in the soda saline–alkali wetland soil occurs at 1.09 PV. Kurwadkar also found that the breakthrough of Br^−^ in farmland soils near the concentrated animal feeding operations in Iowa, Missouri, and North Carolina of the United States occurs within 1–2 PV [38]. The average pore water velocity (v), dispersivity (λ), and dispersion coefficient (D) presented in Table 2 reflect convection-dominated transport with limited dispersion rather than preferential flow characteristics. These characteristics are closely related to the mechanical composition of the soda saline–alkali wetland soil, which has a high proportion of sand particles (51.56%) and a low proportion of clay particles (2.83%). The Br^−^ migration characteristics provide benchmark parameters for subsequent SDZ migration experiments. In summary, the solute transport in the soda saline–alkali wetland soil is mainly dominated by convection, with weak dispersion.

### 2.3. SDZ Migration Characteristics Under Different Cu and Zn Concentrations

**Figure 3:**
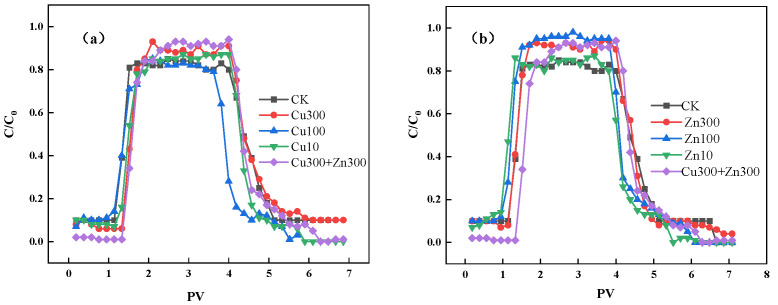
Breakthrough curves of sulfadiazine in soil column experiments under different Cu and Zn treatments: (**a**) Breakthrough curves under different Cu treatments; (**b**) Breakthrough curves under different Zn treatments.

The relatively low organic matter content and the high sand particle proportion of the soda saline–alkali wetland soil led to a relatively high SDZ leaching rate, and the breakthrough occurred between 1.18 and 1.57 PV. The sandy texture and low organic matter content of the soda saline–alkali wetland soil result in limited aggregation and a low density of stable adsorption domains, which weaken SDZ retention. As a consequence, solute transport is dominated by convection with limited diffusion into immobile or strongly sorbing regions, leading to rapid breakthrough and high mobility. Under these conditions, changes in surface chemistry induced by Cu and Zn become a dominant control on SDZ transport behavior. As a result, this indicates a wider migration range than expected for soils with stronger adsorption capacity (Figure 3). Compared with CK, the SDZ breakthrough in Cu- and Zn-treated columns was delayed by approximately 0.25 PV, demonstrating that heavy metals modify transport kinetics rather than merely changing adsorption intensity. Peak concentrations appeared at ~2 PV for all treatments; however, the magnitude and symmetry of the peaks differed due to metal-dependent adsorption kinetics. After 3 PV of SDZ pulse input, leaching with 0.01 mol L^−1^ CaCl_2_ gradually removed SDZ, with full elution by ~6 PV, confirming SDZ as a weakly retained solute under these soil conditions. Cu300 significantly accelerated SDZ migration, yielding a peak relative concentration of 0.93. This behavior is consistent with the reduced distribution coefficient K_d_ (0.285) and the high fraction of instantaneous sorption sites (f = 0.554) shown in the two-site model. Under high Cu loading, the total sorption capacity of the soil decreases, and the remaining sorption is dominated by relatively weak, fast-equilibrating sites, which leads to lower overall retardation and faster breakthrough of SDZ. In contrast, Zn treatments displayed a distinct pattern: Zn10 and Zn100 moderately slowed migration due to enhanced adsorption, while Zn300 accelerated migration due to reduced adsorption strength. Coexisting Cu300 + Zn300 further increased SDZ mobility, producing a peak of 0.94, indicating a nonlinear synergistic enhancement of solute transport, indicating that the weakly adsorbable SDZ is prone to leaching and migration in the soda saline–alkali wetland soil at high Cu and Zn contents, with almost no retention in the soil column. Because the soil pH (~7.85) is high, SDZ predominantly exists as an anion, while Cu^2+^/Zn^2+^ undergo hydroxylation; these pH-dependent processes jointly regulate SDZ adsorption, complexation, and migration. The breakthrough curve shown in Figure 3 also indicates that the adsorption and migration rate of the sulfonamide group of SDZ are related to pH [39,40]. Similar transport behavior has also been reported for other sulfonamides (e.g., sulfamethoxazole) modeled with HYDRUS-1D using non-equilibrium/two-site sorption concepts, suggesting that kinetic retention and site heterogeneity can strongly influence breakthrough behavior in column systems. Moreover, recent studies have highlighted that macropores and preferential flow in undisturbed soils can further accelerate antibiotic transport, implying that packed-column results primarily represent matrix-flow-dominated conditions and should be interpreted as effective laboratory-scale behavior.

**Figure 4:**
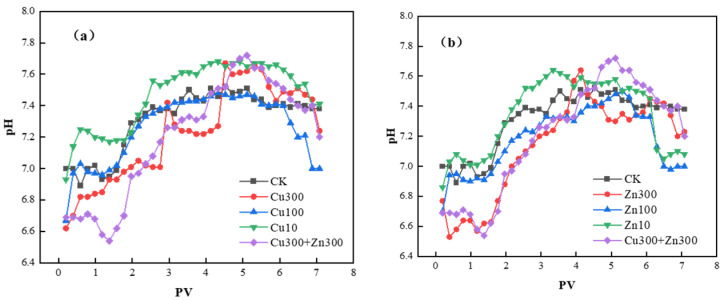
Variation of effluent pH during SDZ leaching under Cu and Zn treatments: (**a**) Effluent pH variation under different Cu treatments; (**b**) Effluent pH variation under different Zn treatments.

The results of soil column breakthrough experiments under different Cu and Zn concentrations are consistent with the batch adsorption experiment results. In the soil with a higher K_f_ (reflecting its SDZ adsorption capacity), the equilibrium SDZ concentration in the effluent of the packed column is lower. Thus, the SDZ migration behavior in the soda saline–alkali wetland soil is closely related to its adsorption characteristics. In the soil column leaching experiment, the effluent pH of all treatments increased initially and then decreased, consistent with a two-stage pH regulation mechanism driven by soil alkalinity and heavy-metal hydrolysis. (Figure 4). The initial rise in pH resulted from the rapid release of base cations (Na^+^, K^+^) and partial desorption of OH^−^ from colloidal surfaces, reflecting the intrinsic alkalinity of the soil. Simultaneously, Cu^2+^ and Zn^2+^ underwent hydrolysis, generating hydroxyl species; this process contributed additional OH^−^ and reinforced the early pH increase [41,42]. The leaching loss of base ions with continuous leaching weakens the soil’s buffering capacity. At higher metal loadings (Cu300, Zn300), accumulated hydroxide precipitates gradually dissolved during continuous leaching, releasing H^+^ and initiating the downward pH shift. SDZ (pKa ≈ 6.5), present predominantly as an anion, formed soluble metal–SDZ complexes that further consumed OH^−^ and contributed to acidification. When Cu and Zn coexisted, their combined complexation intensified proton release, producing a sharper pH decline than single-metal treatments. Without heavy metals (e.g., Cu and Zn) to compete with SDZ in the CK treatment, fewer H^+^ are released, leading to slight pH changes. In contrast, treatments with high heavy metal concentrations feature adsorption site competition and dynamic precipitation changes, exhibiting pronounced pH fluctuations. Thus, the observed pH trend reflects a dynamic interplay of alkaline ion release, metal hydrolysis, precipitation–dissolution processes, and SDZ–metal complexation.

### 2.4. Changes in SDZ Breakthrough Curve Parameters Under Different Cu and Zn Concentrations

Table 3 presents the Hydrus-1D two-site model fitting results, which quantify how Cu and Zn regulate SDZ migration via instantaneous (f, the fraction of instantaneous sorption sites) and kinetic adsorption pathways. The good data fitting of all treatments (R^2^ ≥ 0.915, RMSE < 0.1) verifies the applicability of the two-site model to the SDZ migration behavior in the soda saline–alkali wetland soil. The Hydrus-1D parameters obtained in this study should be interpreted as effective parameters under the specific experimental conditions, rather than unique field-scale constants. Although a formal sensitivity analysis was not performed, the optimized parameters produced stable and consistent simulations of the breakthrough curves across different Cu and Zn treatments. Compared with the inert tracer, the SDZ breakthrough curve is asymmetric, with the peak broadened or narrowed to a certain extent. This phenomenon is unlikely to be ascribed to the hydrodynamic characteristics of the soil column, given the tracer’s relatively ideal breakthrough curve. Thus, the SDZ migration in the soda saline–alkali soil may be hindered or promoted to a certain extent. The differences in the model parameters (f, K_d_, and α) reveal the influence mechanism of heavy metal concentrations on SDZ migration and are closely related to the physicochemical properties of the soil. For the Cu treatments, the fraction of instantaneous sorption sites (f) first decreases from 0.217 (Cu10) to 0.175 (Cu100), and then sharply increases to 0.554 at Cu300. The decrease in f from Cu10 to Cu100 indicates that, as Cu concentration rises within the low–medium range, a larger proportion of SDZ is retained at kinetic sorption sites, which is consistent with the relatively high K_d_ values (0.645 and 0.397) and the enhanced retardation of SDZ transport. At 300 mg kg^−1^ Cu, however, the total sorption capacity declines (K_d_ = 0.285, lower than CK = 0.318), while the remaining sorption is dominated by fast-equilibrating sites (high f). This combination of reduced sorption strength and dominance of weak instantaneous sites leads to earlier breakthrough and higher peak concentrations of SDZ in the Cu300 column. The combined influence of high soil pH (7.85), low organic matter (7.20 g kg^−1^), and metal hydrolysis supports this shift toward kinetic desorption and faster solute transport. Under a high pH, Cu^2+^ is prone to hydrolyze to form Cu(OH)_2_ precipitation, which covers the soil particle surface and alters its charge distribution, leading to adsorption site competition and reorganization. Under the Cu concentration of 10–100 mg kg^−1^, the formation of Cu–SDZ complexes increases total adsorption but does not increase f, meaning adsorption is enhanced primarily through kinetic rather than instantaneous pathways. At a Cu concentration of 300 mg kg^−1^, the saturation of sorption sites and the development of Cu hydroxide precipitates markedly diminish SDZ–soil binding, which is reflected in a decrease in K_d_ from 0.645 to 0.283 and results in enhanced solute mobility. As a result, the SDZ adsorption is inhibited (K_d_ decreases from 0.645 to 0.283), and its migration resistance is decreased. In addition, the soil’s mechanical composition with a high sand particle proportion (51.56%) intensifies the preferential flow effect of solute transport, further accelerating the leaching loss of SDZ under high Cu concentrations.

For the Zn treatments, f increases markedly from 0.101 (Zn10) to 0.267 (Zn100) and 0.553 (Zn300), while K_d_ shows a decreasing trend from 0.373 to 0.213 and 0.230. At the low concentration of 10 mg kg^−1^, the relatively high K_d_ and low f indicate that SDZ is mainly retained at kinetic sorption sites, and overall sorption is still stronger than in the control, which slows down SDZ migration. As Zn concentration increases, the dominance of instantaneous sorption sites (higher f) is accompanied by a reduction in sorption strength (lower Kd), so that SDZ can desorb and move more easily with the percolating solution. Thus, low Zn levels slightly enhance SDZ retention, whereas high Zn levels promote SDZ mobility by weakening the overall sorption capacity of the soil. The high mobility of Zn in the soda saline–alkali soil may be related to its low cation exchange capacity (4.54 cmol kg^−1^) and weak adsorption ability. Under alkaline conditions, Zn^2+^ preferentially combines with dissolved organic carbon (95.02 mg kg^−1^) to form soluble complexes instead of directly competing for adsorption sites, thus increasing SDZ mobility [43]. In addition, the formation of Zn(OH)_2_ may further weaken the interaction between SDZ and soil particles by altering the soil pore structure. As a result, the peak value of the SDZ breakthrough curve is increased, and SDZ is almost completely leached out from the soil column. However, the K_d_ (0.373) at a relatively low Zn concentration (10 mg kg^−1^) is significantly higher than that under high concentrations, indicating that SDZ adsorption is still dominant at this time. This may be because the low-concentration Zn^2+^ is adsorbed on the soil surface through electrostatic attraction, enhancing the coordination adsorption of SDZ, while high-concentration Zn disrupts the adsorption equilibrium through ion exchange [44]. According to the assumption of the two-site model, f is the proportion of the pollutant adsorbed at the “instantaneous adsorption sites” to the total adsorbed amount, and the remaining proportion undergoes kinetic adsorption (e.g., pore diffusion and slow binding). The proportion of instantaneous adsorption sites (f) reflects the kinetic differentiation characteristics of the pollutant adsorption process in the soil. In this study, the f value varied significantly as the heavy metal concentration increased (Table 3). As the dissolved organic carbon in the soil (DOC = 95.02 mg kg^−1^) forms soluble complexes with Zn^2+^, the direct competition for instantaneous adsorption sites with Zn is reduced. Thus, the soil maintains a relatively high f value (0.553) even at a high Zn concentration (300 mg kg^−1^). The f value (0.292) with coexisting Cu and Zn is between those with single heavy metals, but the K_d_ value (0.205) is significantly lower than that of the blank group (0.318). These results indicate complex synergistic effects of the two heavy metals on SDZ migration. The two metal ions may coprecipitate and form new surface active sites to partially offset the inhibitory effect of a single heavy metal [45]. The low organic matter content of the soda saline–alkali soil further exacerbates the instability of this dynamic equilibrium, contributing to the nonlinearity of the SDZ migration behavior.

The SDZ migration behavior in the soil under dynamic conditions was studied through soil column leaching experiments. The results suggest that the adsorption coefficient (K_d_) derived from soil column leaching experiments is not always consistent with the results of batch equilibrium adsorption experiments. The batch experiment results are generally higher than the K_d_ values from column experiments, suggesting that the batch experiment conditions may overestimate the adsorption capacity. This difference can be explained by the different contact time and the continuous flow conditions within the column, and the soil column experiments are closer to the natural environmental conditions than the batch experiments. This discrepancy arises because batch adsorption experiments are conducted under static conditions with continuous shaking, long contact times, and full soil–solution mixing, which promote near-equilibrium sorption and maximize access to adsorption sites. In contrast, soil column experiments operate under dynamic flow conditions, where solute–soil contact time is limited and adsorption is often kinetically constrained, particularly in sandy soils with weak diffusion domains. As a result, column-derived K_d_ values more directly reflect transport-controlled retention and tend to be lower than those obtained from batch systems [46].

Changes in the two-site model parameters reflect the heterogeneity of the soda saline–alkali wetland soil and the characteristics of its adsorption kinetics. The high sand proportion (51.56%) and low clay proportion (2.83%) lead to a weak water-holding capacity, with solute transport dominated by convection and a weak dispersion effect (Table 3, λ = 0.176 cm). These findings are consistent with the results of the Br^−^ tracer experiment (Figure 2). The low organic carbon content (7.20 g kg^−1^) also constrains the hydrophobic adsorption of SDZ by the soil, making the competitive adsorption of heavy metals the dominant mechanism. The special physicochemical properties of the soda saline–alkali wetland soil (high pH, low organic matter, sandy structure) and the concentration-dependent effects of heavy metals jointly determine the SDZ migration behavior, similar to the research results of Zhang [47]. Despite the valuable insights obtained from this study, several limitations should be briefly acknowledged. The soil column experiments were conducted using laboratory-packed columns under controlled conditions, which mainly represent matrix-flow-dominated transport and do not fully capture field-scale heterogeneity such as macropores and preferential flow pathways commonly present in natural wetlands. In addition, the Hydrus-1D model employed in this study provides a one-dimensional description of solute transport, and the fitted parameters should be interpreted as effective parameters specific to the experimental conditions, rather than universal constants applicable to field systems. Nevertheless, this combined experimental–modeling approach is appropriate for quantitatively comparing concentration-dependent trends and for elucidating the influence of Cu and Zn on SDZ migration under well-defined conditions.

## 3. Materials and Methods

### 3.1. Chemical Reagents

Sulfadiazine (SDZ, purity ≥ 98.0%) and potassium bromide (KBr, purity ≥ 99.0%) were purchased from Shanghai Macklin Biochemical Co., Ltd. (Shanghai, China). Copper nitrate trihydrate (Cu(NO_3_)_2_·3H_2_O, purity ≥ 98.0%) and zinc chloride (ZnCl_2_, purity ≥ 98.0%) were obtained from Sinopharm Chemical Reagent Co., Ltd. (Shanghai, China). Quartz sand (40–60 mesh) was also supplied by Shanghai Macklin Biochemical Co., Ltd. (Shanghai, China). All chemicals used in this study were of analytical grade. Ultrapure water was used for the preparation of all solutions.

### 3.2. Soil Sample Collection and Preparation

The soil samples for the experiment were collected in July 2024 from Niuxintao’bao National Wetland Park (45°13′–45°16′ N, 123°13′–123°21′ E) in Da’an City, Jilin Province, China. Soil sampling was conducted once during the field campaign. Approximately 15 kg of soil was collected using a five-point composite sampling method, in which subsamples were taken from evenly distributed locations within the reed wetland and combined to form a representative bulk soil sample. This composite sampling strategy was adopted to reduce spatial heterogeneity and ensure that the collected soil adequately represented the physicochemical characteristics of the study area. This region is characterized by a continental semi-humid and arid monsoon climate zone in the middle temperate zone. The average annual precipitation is 400–450 mm, with evaporation significantly exceeding precipitation. Notably, precipitation from June to August constitutes over 70% of the annual total, resulting in pronounced seasonal droughts. Surface water in this area predominantly exhibits alkaline properties. The soil in the study area is a salinized soil of the NaHCO_3_ type, showing moderate salinization and alkalization characteristics. It is further characterized by poor permeability and high base saturation, representing a typical soda saline–alkali reed wetland ecosystem.

In the area where the reed community was evenly distributed, soil samples from the middle layer (at a depth of 20–40 cm) were collected using the “five-point mixed sampling method”. After removing plant roots and gravel, the samples were put into sealed bags. Upon transportation to the laboratory, they were spread out in a cool and ventilated place to air-dry naturally. Subsequently, the samples were gently pulverized with a wooden mallet to ensure dispersion and passed through a 2 mm nylon sieve for further processing. After homogenization, the soil samples were sealed and stored for subsequent analysis (Table 4).

Briefly, 5.0000 g (±0.0005 g) of the soil sample was weighed; then, 25 mL of ultrapure water was added, shaken for 30 min, and then allowed to settle. The pH of the soil was measured using a pH meter (PHS-3C, Lei Ci, Shanghai, China). The electrical conductivity (EC) was measured using the electrode method. The sodium acetate exchange method determined the cation exchange capacity (CEC). The content of soluble salts was determined by the gravimetric method following oxidizing and digesting with H_2_O_2_ until a constant weight was achieved at a soil-to-water ratio of 1:5. The organic carbon and total nitrogen were determined using a carbon-nitrogen elemental analyzer (vario MACRO cube, Elementar, Langenselbold, Germany). The dissolved organic carbon was detected by a total organic carbon analyzer (TOC-LCPH, Shimadzu, Kyoto, Japan). The soil mechanical composition was characterized by a laser particle size analyzer (MASTERSIZER 2000, Malvern, Worcestershire, UK).

In the experiment, a gradient treatment of combined pollution of Cu and Zn was set up. Stock solutions of 1000 mg L^−1^ were prepared using Cu(NO_3_)_2_·3H_2_O and ZnCl_2_, respectively. An appropriate amount of the stock solutions was accurately pipetted and diluted with ultrapure water to achieve the desired concentration gradients (0, 10, 100, 300 mg kg^−1^). The diluted solutions were added dropwise to 400 g of soil and stirred thoroughly with a glass rod to ensure the homogeneity of the soil-solution mixture. The specific treatments of the eight samples were as follows: (1) CK, a blank control without the addition of heavy metals; (2) Cu10, with a Cu addition of 10 mg kg^−1^ in the soil; (3) Cu100, with a Cu addition of 100 mg kg^−1^ in the soil; (4) Cu300, with a Cu addition of 300 mg kg^−1^ in the soil; (5) Zn10, with a Zn addition of 10 mg kg^−1^ in the soil; (6) Zn100, with a Zn addition of 100 mg kg^−1^ in the soil; (7) Zn300, with a Zn addition of 300 mg kg^−1^ in the soil; (8) Cu300 + Zn300, with a Cu content of 300 mg kg^−1^ and a Zn content of 300 mg kg^−1^ in the soil. All soil samples were air-dried under natural conditions, sieved through a 2 mm mesh, and equilibrated in the dark for seven days before use. The selected Cu and Zn concentrations were designed to represent a gradient from low to high contamination scenarios. The lower levels (10 and 100 mg kg^−1^) fall within the range reported for soils influenced by agricultural activities, whereas the highest level (300 mg kg^−1^) was included as an upper-bound experimental scenario to systematically examine concentration-dependent effects on sulfadiazine adsorption and migration. This concentration was not intended to directly simulate average field conditions, but to support a comprehensive assessment of metal interference under severe contamination scenarios.

### 3.3. Batch Equilibrium Adsorption Experiment

Briefly, 5.0000 g (±0.0005 g) of the soil sample was weighed and transferred to a 50 mL polypropylene centrifuge tube. Using a 0.01 mol L^−1^ CaCl_2_ solution as the background electrolyte, SDZ solutions with concentration gradients of 10, 15, 20, 25, and 30 mg L^−1^ were prepared and a blank group was set up. After adjusting the pH of all solutions to 7.00 ± 0.05 using 0.1 mol L^−1^ HCl and NaOH solutions, 10 mL of SDZ solutions of different concentrations was added according to the water-to-soil ratio of 2:1 (V/m). The mixed system was placed in a constant-temperature oscillator at 25 ± 0.5 °C and shaken at 250 r min^−1^ for 24 h. Following centrifugation at 4000 r min^−1^ for 5 min, the supernatant was collected and filtered through a 0.22 μm membrane. The concentration of sulfadiazine in the supernatant was determined using a Shimadzu LC-20 high-performance liquid chromatography (HPLC) system equipped with an ultraviolet (UV) detector. The chromatographic operating conditions were as follows: a C18 chromatographic column (4.6 mm × 150 mm) was used, with a flow rate of 1 mL min^−1^ and the column temperature was controlled at 30 °C. The detection wavelengths were set at 270 nm, the detection time was set to 10 min, and the injection volume was 10 μL. The mobile phase was a mixture of acetonitrile and water at a volume ratio of 20:80. The retention time of sulfadiazine was 2.67 min (R^2^ = 0.99997).

The adsorption amount of SDZ in the soil can be calculated as follows:

(1) $$q_{e}=\frac{\left(C_{0}-C_{t}\right)V}{m}$$

where *V* is the volume of the solution (mL); *C*_0_ and *C_t_* are the initial concentration of SDZ and the concentration of the solution at time *t* (mg L^−1^), respectively; *m* represents the mass (g).

Common batch equilibrium isothermal adsorption models include the Freundlich and Langmuir equations, which are expressed as Equation (2) and Equation (3), respectively.

(2) $$lnq_{e}=lnk_{f}+\frac{1}{nlnC_{e}}$$

(3) $$C_{e}/q_{e}=C_{e}/q_{max}+\frac{1}{q_{max}K_{L}}$$

where *C_e_* denotes the mass concentration of SDZ in the soil solution at the adsorption or desorption equilibrium state (mg L^−1^); *q_e_* denotes the mass concentration of SDZ in the soil at the adsorption or desorption equilibrium state (mg kg^−1^); *q_max_* represents the saturated adsorption capacity (mg kg^−1^); *K_f_* is the adsorption constant of the Freundlich equation; *K_L_* is the adsorption constant of the Langmuir model; *n* is the adsorption index of the Freundlich equation.

### 3.4. Soil Column Experiment

This experiment employed a plexiglass column to simulate the pollutant migration process. The plexiglass column measured 15.0 cm in length and had an inner diameter of 5.0 cm. A total of 400.0 g of air-dried soil samples were packed in layers according to the field bulk density of 1.34 g cm^−3^, and mechanical compaction was performed after each 3 cm layer was filled to ensure uniform packing. Subsequently, absorbent cotton was placed at both ends of the column to prevent soil particles from leaking. Then, a quantitative filter paper with a matching diameter and a 2 cm thick pre-treated quartz sand layer was laid. The pre-treatment method for the quartz sand involved soaking it in 1 mol L^−1^ HCl for 24 h, followed by repeated rinsing with ultrapure water until neutrality was achieved. Finally, the quartz sand was dried in an oven at 105 °C to ensure uniform infiltration of the solution. A 0.01 mol L^−1^ CaCl_2_ solution was injected from the bottom upwards at a flow rate of 0.3 mL min^−1^ using a peristaltic pump (BT-100F, Baoding Longer, Baoding, China) to continuously moisten the soil column for more than 24 h. The soil column was considered to have reached a saturated and stable state when the fluctuation range of pH and EC in the effluent was less than 5%.

#### 3.4.1. Br^−^ Tracer

A Br^−^ solution with a concentration of 100 mg L^−1^ (the solvent is 0.01 mol L^−1^ CaCl_2_ and pH = 7) was pulse-injected into the soil column at a flow rate of 0.6 mL min^−1^ for 5 pore volumes (PV = porosity × volume of the soil column). Upon completion of the Br^−^ injection, the Br^−^ solution was switched to a pure CaCl_2_ solution for leaching. The filtrate was collected using an automatic collector (BSZ-40, Huxi, Shanghai, China). The concentration of Br^−^ in the effluent was monitored by an ion chromatograph (Dionex ICS-1100, ThermoFisher, Waltham, MA, USA) until Br^−^ was no longer detectable. Then, the breakthrough curve was obtained, and the Hydrus-1D software was used for inverse modeling to analyze the hydraulic properties of the soil column.

#### 3.4.2. SDZ Migration Experiment

A 30.0 mg L^−1^ SDZ solution (pH = 7.0, with the same solvent as above) was used to replace the existing solution. After pulse-injecting 3 PV at the same flow rate, leaching was carried out using a pure CaCl_2_ solution until no SDZ was detected in the effluent.

#### 3.4.3. Solute Transport Model

The classical Convection–Dispersion Equation (CDE) usually describes the migration of solutes, which can be expressed by Equations (4) and (5):

(4) $$\frac{\partial c}{\partial t}=\frac{D}{r}\frac{\partial^{2}c}{\partial x^{2}}-\frac{v}{r}\frac{\partial c}{\partial x}$$

(5) $$r=1+\frac{\rho K_{d}}{\theta_{v}}$$

where *c* represents the solute concentration, measured in mg L^−1^; *t* stands for time (hour); *D* denotes the hydrodynamic dispersion coefficient (cm^2^ h^−1^); *r* is the retardation factor; *x* represents the distance (cm); *v* represents the average pore-water velocity (cm h^−1^); *ρ* is the dry bulk density of the soil (g cm^−3^); *θ_v_* is the volumetric water content (cm^3^/cm^3^). These parameters are fundamental in the context of the equations used to describe relevant processes.

Because the adsorption process of SDZ on the soil is kinetically limited, its reactive migration can be described based on a two-site model with Freundlich kinetic adsorption, which can be expressed by Equations (6)–(8):

(6) $$\left(1+\frac{f\rho K_{d-c}}{\theta_{v}}\right)\frac{\partial c}{\partial t}+\frac{\rho }{\theta_{v}}\frac{\partial s_{2}}{\theta_{t}}=D\frac{\partial^{2}c}{\partial x^{2}}-v\frac{\partial c}{\partial x}$$

(7) $$\frac{\partial s_{1}}{\theta_{t}}=fK_{d-c}\frac{\partial c}{\partial t}$$

(8) $$\frac{\partial s_{2}}{\partial t}=\left[\left(1-f\right)K_{d-c}c-s_{2}\right]$$

where *f* signifies the fraction of the instantaneous adsorption exchange at the equilibrium state; *s*_1_ represents the adsorption concentration at the instantaneous adsorption site (mg kg^−1^); *s*_2_ stands for the adsorption concentration at the kinetic adsorption site (mg kg^−1^); *α* denotes the first-order kinetic rate coefficient (h^−1^); *K_d-c_* is the instantaneous adsorption constant within the two-site model (L kg^−1^). These parameters play crucial roles in the context of the relevant equations for describing the related adsorption and migration processes.

The correlation coefficient (R^2^) and the root mean square error (RMSE) were adopted as the determination indices to quantify the degree of coincidence between the simulated and observed values. A value of R^2^ closer to 1 indicates a higher model fitting accuracy, while a smaller RMSE signifies reduced prediction error.

### 3.5. Data Processing

All the measured data were processed, calculated, and subjected to statistical analysis using Microsoft Excel 2020 software. The adsorption and migration processes of SDZ in the soil were simulated using Origin 2021 software and Hydrus-1D software (4.17.0140.exe, US), Manufacturer: USDA-ARS U.S. Salinity Laboratory, Riverside, CA 92507, USA. respectively.

## 4. Conclusions

This study demonstrated that Cu and Zn co-contamination significantly alters the adsorption and migration behavior of sulfadiazine (SDZ) in a typical soda saline–alkali wetland soil. Batch adsorption and soil column experiments showed that increasing metal concentrations enhanced SDZ retention while simultaneously modifying its transport dynamics, resulting in clear concentration-dependent effects under alkaline–saline conditions. The Hydrus-1D model successfully reproduced the breakthrough curves and captured the influence of metal presence on SDZ migration.

The results further indicate that metal–antibiotic interactions play a key role in controlling SDZ mobility in saline–alkali soils characterized by high pH and weak adsorption capacity. The model-derived parameters quantitatively describe SDZ transport behavior and suggest that coexisting Cu and Zn can regulate adsorption site distribution and delay SDZ migration, emphasizing the importance of considering heavy metals when interpreting antibiotic fate in wetland environments.

Overall, this work provides quantitative evidence for understanding antibiotic transport under combined metal contamination and contributes to improved assessment of migration risks in soda saline–alkali wetlands. The experimental data and modeling framework can be applied to support environmental risk assessment and management of wetlands receiving agricultural drainage or metal-contaminated inputs. Future research should focus on field-scale validation, incorporation of soil structural heterogeneity and preferential flow, and extension of this approach to other antibiotics with different physicochemical properties.

## Figures and Tables

**Table 1:** The fitting parameters of the isothermal adsorption model of sulfadiazine in the soda saline–alkali wetland soils.

Experimental Treatment	Freundlich Equation			Langmuir Equation		
	K_f_	1/n	R^2^	K_L_	q_max_	R^2^
CK	0.181	0.952	0.980	0.004	40.978	0.955
Cu10	0.156	1.015	0.980	NA	NA	NA
Cu100	0.253	0.887	0.984	0.008	26.138	0.972
Cu300	0.168	0.902	0.990	0.044	7.041	0.944
Zn10	0.232	0.916	0.987	0.006	32.901	0.972
Zn100	0.276	0.851	0.995	0.010	20.870	0.990
Zn300	0.206	0.904	0.977	0.007	24.197	0.948
Cu300 + Zn300	0.175	0.863	0.922	0.010	13.167	0.820

Note: CK, control; Cu10 treatment, Cu 10 mg kg^−1^; Cu100 treatment, Cu 100 mg kg^−1^; Cu300 treatment, Cu 300 mg kg^−1^; Zn10 treatment, Zn 10 mg kg^−1^; Zn100 treatment, Zn 100 mg kg^−1^; Zn300 treatment, Zn 300 mg kg^−1^; Cu300 + Zn300 treatment, Cu 300 mg kg^−1^ and Zn 300 mg kg^−1^. NA: NA did not converge.

**Table 2:** Hydraulic parameters were inverted based on BTCs of Br^−^ in the tested soil column.

Parameters	v (cm h^−1^)	λ (cm)	D (cm^2^/h)	R^2^	RMSE
Soil column	4.370	0.176	0.770	0.900	0.039

Note: The v is the average pore flow rate, λ is the dispersivity, D is the dispersion coefficient, R^2^ is the correlation coefficient, and RMSE is the root-mean-square error.

**Table 3:** Fitting parameters of sulfadiazine with BTCs in the presence of Cu/Zn.

Experimental Treatment	f	K_d_	α	R^2^	RMSE
CK	0.292	0.318	0.001	0.996	0.033
Cu10	0.217	0.645	0.006	0.977	0.029
Cu100	0.175	0.397	0.005	0.915	0.062
Cu300	0.554	0.285	0.003	0.970	0.032
Zn10	0.101	0.373	0.001	0.979	0.027
Zn100	0.267	0.213	0.002	0.973	0.025
Zn300	0.553	0.230	0.002	0.986	0.024
Cu300 + Zn300	0.292	0.205	0.027	0.963	0.038

Note: CK, control; Cu10 treatment, Cu 10 mg kg^−1^; Cu100 treatment, Cu 100 mg kg^−1^; Cu300 treatment, Cu 300 mg kg^−1^; Zn10 treatment, Zn 10 mg kg^−1^; Zn100 treatment, Zn 100 mg kg^−1^; Zn300 treatment, Zn 300 mg kg^−1^; Cu300 + Zn300 treatment, Cu 300 mg kg^−1^ and Zn 300 mg kg^−1^.

**Table 4:** Physical and chemical properties of soda saline–alkali wetland soils.

Parameters	Soda Saline–Alkali Wetland Soils
pH	7.85 ± 0.10
EC (μS cm^−1^)	172.10 ± 0.77
Content of Soluble Salts (g kg^−1^)	1.10 ± 0.10
CEC (cmol kg^−1^)	4.54 ± 0.40
OC (g kg^−1^)	7.20 ± 0.08
DOC (mg kg^−1^)	95.02 ± 2.87
clay proportion (%)	2.83 ± 0.10
silt proportion (%)	45.61 ± 0.20
sand proportion (%)	51.56 ± 0.10

Note: EC stands for electrical conductivity, CEC represents cation exchange capacity, OC refers to organic carbon, DOC means dissolved organic carbon, and clay proportion, silt proportion and sand proportion are components of soil mechanical composition.

## Data Availability

The data presented in this study are available on reasonable request from the corresponding author when necessary.

## References

[1] Gaballah M.S., Guo J., Sun H., Aboagye D., Sobhi M., Muhmood A., Dong R. (2021). A review targeting veterinary antibiotics removal from livestock manure management systems and future outlook. Bioresour. Technol..

[2] Gros M., Marti E., Balcázar J.L., Boy-Roura M., Busquets A., Colón J., Sànchez-Melsió A., Lekunberri I., Borrego C.M., Ponsá S. (2019). Fate of pharmaceuticals and antibiotic resistance genes in a full-scale on-farm livestock waste treatment plant. J. Hazard. Mater..

[3] Yan X.-T., Zhai Y.-Q., Cai Y.-Y., Guo Z., Zhang Q.-Q., Ying G.-G. (2022). Hypothetical scenarios estimating and simulating the fate of antibiotics: Implications for antibiotic environmental pollution caused by farmyard manure application. Sci. Total Environ..

[4] Zhang Q.Q., Ying G.G., Pan C.G., Liu Y.S., Zhao J.L. (2015). Comprehensive evaluation of antibiotics emission and fate in the river basins of China: Source analysis, multimedia modeling, and linkage to bacterial resistance. Environ. Sci. Technol..

[5] Andreu V., Gimeno-García E., Pascual J., Vazquez-Roig P., Picó Y. (2016). Presence of pharmaceuticals and heavy metals in the waters of a Mediterranean coastal wetland: Potential interactions and the influence of the environment. Sci. Total Environ..

[6] (2020). Environmental Pollution; Study Data from University of Jinan Update Knowledge of Environmental Pollution (The presence of tetracyclines and sulfonamides in swine feeds and feces: Dependence on the antibiotic type and swine growth stages). Ecol. Environ. Conserv..

[7] Ovung A., Bhattacharyya J. (2021). Sulfonamide drugs: Structure, antibacterial property, toxicity, and biophysical interactions. Biophys. Rev..

[8] Chen K.L., Liu L.C., Chen W.R. (2017). Adsorption of sulfamethoxazole and sulfapyridine antibiotics in high organic content soils. Environ. Pollut..

[9] Guo X., Shen X., Zhang M., Zhang H., Chen W., Wang H., Koelmans A., Cornelissen G., Tao S., Wang X. (2017). Sorption mechanisms of sulfamethazine to soil humin and its subfractions after sequential treatments. Environ. Pollut..

[10] Ma N., Zhang H., Yuan L., Li Y., Yang W., Huang Y. (2024). Biotransformation of enrofloxacin-copper combined pollutant in aqueous environments by fungus Cladosporium cladosporioides (CGMCC 40504). World J. Microbiol. Biotechnol..

[11] Tang T., Yang C., Wang L., Jiang X., Dang Z., Huang W. (2018). Complexation of sulfamethazine with Cd(II) and Pb(II): Implication for co-adsorption of SMT and Cd(II) on goethite. Environ. Sci. Pollut. Res. Int..

[12] Yang L.-L., Lin M., Wei L., Yi-Fan C., Qi-Yang T., Qiao-Hong Z., Zhen-Bin W., Feng H. (2022). Purification Performance of Rural Livestock and Poultry Breeding Tail Water by Constructed Wetland Under Cu and SMZ Combined Pollution. Acta Hydrobiol. Sin..

[13] Zhao K., Li C., Wang Q., Lu H. (2022). Distribution of Sulfonamide Antibiotics and Resistance Genes and Their Correlation with Water Quality in Urban Rivers (Changchun City, China) in Autumn and Winter. Sustainability.

[14] Jin C., Wei S., Sun R., Zou W., Zhang X., Zhou Q., Liu R., Huang L. (2020). The Forms, Distribution, and Risk Assessment of Sulfonamide Antibiotics in the Manure-Soil-Vegetable System of Feedlot Livestock. Bull. Environ. Contam. Toxicol..

[15] Zhang Y., Hao W., Liu S., Ren S., Min J., Zou C. (2026). Distribution patterns of antibiotic resistance genes in Chinese vegetable farmlands and key soil physicochemical factors. J. Environ. Chem. Eng..

[16] Chen L., Meng X., Zhou X., Wang J., Fu Y., Wang S., Ma Y., Shen Z. (2025). New insights into the distribution and risk of antibiotics: From point to non-point source in a rapidly urbanizing watershed—A case study of the Wenyu River, Beijing. J. Clean. Prod..

[17] Li B., Gao H., Li R., Jia X., Yao W., Yao Z. (2026). Characteristics of regionalized distribution of antibiotics and ARGs in Daliao River-Liaodong Bay waters and their environmental impact factors. J. Environ. Sci..

[18] Jian M., Che Y., Gao M., Zhang X., Zhang Z., Tan C., Li H. (2024). Migration of naphthalene in a biochar-amended bioretention facility based on HYDRUS-1D analysis. J. Environ. Manag..

[19] Li J., Zhao R., Li Y., Chen L. (2018). Modeling the effects of parameter optimization on three bioretention tanks using the HYDRUS-1D model. J. Environ. Manag..

[20] Zhu S., Liu B., Li S., Zhang L., Rene E.R., Ma W. (2025). Simulation and prediction of sulfamethazine migration, transformation and risk diffusion during cross-media infiltration from surface water to groundwater driven by dynamic water level: Machine learning coupled HYDRUS-GMS model. J. Environ. Manag..

[21] Wu Y., Song S., Li F., Cui H., Wang R., Yang S., Li Z., Chen G. (2023). Multimedia fate of sulfamethoxazole (SMX) in a water-scarce city by coupling fugacity model and HYDRUS-1D model. Sci. Total Environ..

[22] Li J., Feng O., Shijie Z., Xinyu L., Yang H., Shuaifan R. (2022). Effects of Biochar on Sorption and Transport of Florfenicol in Purple Soil. Res. Environ. Sci..

[23] Liu X. (2003). Tactical study on the ecological protection of the western Songnen Plain. SSCSA.

[24] Aslam M.A., Abbas M.S., Mustaqeem M., Bashir M., Shabbir A., Saeed M.T., Irfan R.M. (2024). Comprehensive assessment of heavy metal contamination in soil-plant systems and health risks from wastewater-irrigated vegetables. Colloids Surf. C Environ. Asp..

[25] Li Q., Dai Q., Hu J., Wu H., Chen J. (2022). Profiles of tetracycline resistance genes in paddy soils with three different organic fertilizer applications. Environ. Pollut..

[26] Zhang J. (2011). Adsorption Behavior of Sulfadiazine and Copper in Soil and Its Influencing Factors. Master’s Thesis.

[27] Zhang B.D., Lin Q., Xu S.H. (2018). Effects of Cd/Cu/Pb on Adsorption and Migration of Sulfadiazine in Soil. Acta Pedol. Sin..

[28] Li Y., Wang X., Wang Y., Wang F., Xia S., Zhao J. (2020). Struvite-supported biochar composite effectively lowers Cu bio-availability and the abundance of antibiotic-resistance genes in soil. Sci. Total Environ..

[29] Chen R., Cai B., Liu R., Xu W., Jin X., Yu L., Wang Q., Yong Q. (2024). Synergistic and antagonistic adsorption mechanisms of copper(II) and cefazolin onto bio-based chitosan/humic composite: A combined experimental and theoretical study. J. Environ. Chem. Eng..

[30] Tahervand S., Jalali M. (2017). Sorption and desorption of potentially toxic metals (Cd, Cu, Ni and Zn) by soil amended with bentonite, calcite and zeolite as a function of pH. J. Geochem. Explor..

[31] Hu T., Hao Q., Qian X., Yan G., Gu J., Sun W. (2025). Normal levels of Cu and Zn contamination present in swine manure increase the antibiotic resistance gene abundances in composting products. Process Saf. Environ. Prot..

[32] Wang Z., Sun Y., Wang X., Xia S., Zhao J. (2025). Biochar reversed antibiotic resistance genes spread in biodegradable microplastics and Cu co-contaminated soil by lowering Cu bio-availability and regulating denitrification process. J. Environ. Chem. Eng..

[33] Yuan S., Wang Z., Yuan S. (2024). Insights into the pH-dependent interactions of sulfadiazine antibiotic with soil particle models. Sci. Total Environ..

[34] Xu Y., Yu W., Ma Q., Zhou H. (2015). Interactive effects of sulfadiazine and Cu(II) on their sorption and desorption on two soils with different characteristics. Chemosphere.

[35] Doretto K.M., Rath S. (2013). Sorption of sulfadiazine on Brazilian soils. Chemosphere.

[36] Conde-Cid M., Nóvoa-Muñoz J., Fernández-Sanjurjo M., Núñez-Delgado A., Álvarez-Rodríguez E., Arias-Estévez M. (2019). Pedotransfer functions to estimate the adsorption and desorption of sulfadiazine in agricultural soils. Sci. Total Environ..

[37] Cela-Dablanca R., Barreiro-Buján A., Ferreira-Coelho G., López L.R., Santás-Miguel V., Arias-Estévez M., Núñez-Delgado A., Fernández-Sanjurjo M.J., Álvarez-Rodríguez E. (2022). Competitive adsorption and desorption of tetracycline and sulfadiazine in crop soils. Environ. Res..

[38] Kurwadkar S.T., Adams C.D., Meyer M.T., Kolpin D.W. (2011). Comparative mobility of sulfonamides and bromide tracer in three soils. J. Environ. Manag..

[39] Lertpaitoonpan W., Ong S.K., Moorman T.B. (2009). Effect of organic carbon and pH on soil sorption of sulfamethazine. Chemosphere.

[40] Srinivasan P., Sarmah A.K. (2014). Assessing the sorption and leaching behaviour of three sulfonamides in pasture soils through batch and column studies. Sci. Total Environ..

[41] Liu Z., Han Y., Jing M., Chen J. (2017). Sorption and transport of sulfonamides in soils amended with wheat straw-derived biochar: Effects of water pH, coexistence copper ion, and dissolved organic matter. J. Soils Sediments.

[42] Lyu S., Hu S., Rong F., Gao X., Chao L., Liu A. (2023). Effects of copper on the adsorption—Desorption characteristics of sulfadiazine in soil at different calcium ion concentrations. Environ. Pollut. Control.

[43] Wei M., Wang X., Zhou K., Yang R. (2023). Binary Adsorption and Migration Simulation of Levofloxacin with zinc at Concentrations Simulating Wastewater on Silty Clay and The Potential Environmental Risk in Groundwater. Chemosphere.

[44] Wang S., Wang H. (2015). Adsorption behavior of antibiotic in soil environment: A critical review. Front. Environ. Sci. Eng..

[45] Urdiales C., Urdiales-Flores D., Tapia Y., Caceres-Jensen L., Šimůnek J., Antilén M. (2025). Transport mechanisms of the anthropogenic contaminant sulfamethoxazole in volcanic ash soils at equilibrium pH evaluated using the HYDRUS-1D model. J. Hazard. Mater..

[46] Hamdi S., Mosbahi M., Issaoui M., Barreiro A., Cela-Dablanca R., Brahmi J., Tlili A., Jamoussi F., Fernández-Sanjurjo M.J., Núñez-Delgado A. (2024). Experimental data and modeling of sulfadiazine adsorption onto raw and modified clays from Tunisia. Environ. Res..

[47] Zhang Y., Shao M., Zhang H., Li Y., Liu D., Cheng Y., Liu G., Wang J. (2014). Synthesis and reactivity of oxygen chelated ruthenium carbene metathesis catalysts. J. Organomet. Chem..

